# International comparison of patient-reported outcome and experience measures among people living with mental health conditions

**DOI:** 10.1192/j.eurpsy.2026.10402

**Published:** 2026-07-31

**Authors:** C. Kendir

**Affiliations:** Health Division, OECD, Paris, France

## Abstract

**Abstract:**

Healthcare systems increasingly recognise that lived experience is not an adjunct to evidence, but a core component of it, to increase the value of care for patients and make healthcare systems more people centred. This presentation examines how OECD member countries collect and use patient-reported outcome measures (PROMs) and patient-reported experience measures (PREMs) to assess the performance of healthcare systems in delivering mental health care. Drawing on OECD work on the Patient-Reported Indicator Surveys (PaRIS) and in mental health PROMs and PREMs, the presentation explores how health outcomes and experiences of care can be translated into comparable policy indicators.

The presentation will address the journey from data to evidence through co-production with people with lived experience across indicator development, data collection, and interpretation, rather than confined to one-off consultations. It will also provide an overview of results from the OECD work on the PROMs and PREMs in mental health care and among people living with mental health conditions.

By reflecting on lessons from international measurement initiatives, the presentation will highlight policy and practical implications for policymakers, researchers, practitioners and service users to make healthcare systems more centred to people’s needs.

**Disclosure of Interest:**

None Declared

